# Supplementary material for: Are carbon emissions trading and green financial instruments synergistic? -Comprehensive quantitative research based on content analysis
Source: PLoS One. 2024 Mar 7;19(3):e0298601. doi: 10.1371/journal.pone.0298601 (PMC10919601; doi:10.1371/journal.pone.0298601)
Supplement: S1 Data — (ZIP) [file pone.0298601.s001.zip › Data/地级市Z_F工作报告.zip!/╜¡╦╒/╒≥╜¡╩╨/└┤╫╘╣1⁄2╓┌║┼í╛┬φ┐╦╩2╛▌═°í┐_2016.docx]

各位代表：

　　现在，我代表镇江市人民政府向大会作工作报告，请予审议，并请各位政协委员提出意见。

一、2015年和“十二五”回顾

　　刚刚过去的一年，我们以习近平总书记视察镇江为强大动力，以“镇江很有前途”为巨大激励，在省委、省政府和市委的坚强领导下，全面贯彻党的十八大和十八届三中、四中、五中全会以及习近平总书记系列重要讲话精神，深入落实“四个全面”战略布局，坚持生态领先，推进特色发展，励精图治建设“强富美高”新镇江，圆满完成市七届人大四次会议确定的各项目标任务。

　　（一）以一般公共预算收入突破300亿元为标志，经济发展取得新业绩。全年预计实现地区生产总值3500亿元，增长9.3%；一般公共预算收入302.8亿元，增长9%；固定资产投资2500亿元，增长18%；社会消费品零售总额1110亿元，增长11%；城乡居民人均可支配收入分别达3.9万元和1.9万元，增长8.5%和9.5%。210个市级重点产业项目完成投资810亿元，完成工业技改投资562亿元，产业类实到外资13亿美元，吸引民资310亿元；完成外贸出口68亿美元。规模以上工业企业实现利税864亿元、利润560亿元，分别增长11.2%和11.9%。新增新三板和区域股权交易市场挂牌企业44家，超历年总和；发行直接债务融资工具167.5亿元，居全省前列。全市金融机构存款余额突破4000亿元。政府性债务得到有效管控，债务增长和融资成本实现“双降”，探索破解企业“担保链”，区域金融生态保持良好。

　　（二）以全面落实“励精图治”要求为标志，各地发展呈现新亮点。丹阳市完成新兴产业、服务业、技改 “三个百亿”投入计划，新增上市挂牌企业26家，跻身全国综合实力百强县20强，创成国家循环经济示范市。句容市一般公共预算收入增幅保持全市第一、在全省排名进两位，旅游业实现综合收入150亿元，现代农业、生态建设走在全省前列。扬中市荣登全国县域经济创新力榜首，创成国家卫生城市、国家园林城市，获批筹建省级高新区。丹徒区以北汽项目为带动，孟家港国际物流园、宝龙城市广场等大项目齐头并进。京口区现代服务业集聚区建设成效明显，应税营业收入增幅位列全市第一，引进总部经济及配套企业突破100家。润州区以国家高新区建设为主抓手，新增省级以上高新技术企业24家、省级以上重大创新载体8个。镇江新区国家级综合保税区成功获批并通过验收，引进席勒直升机等一批重大项目，航空航天、新材料产业集聚效应初显，成功举办国际复合材料大会。

　　（三）以北汽华东（镇江）基地首车下线为标志，产业转型实现新突破。坚持不懈推进产业“三集”发展，三类园区的产业集中度分别提高2.4、2.7和2.2个百分点，6个试点园区实行市场化运作。北汽镇江基地345天建成一座现代化工厂，速度成为行业之最；中储粮二期等10个重大产业项目竣工投产。新兴产业实现销售3925亿元，增长10.3%，占全市工业销售的46.2%。苏南国家自主创新示范区确立“一区十四园”发展格局，国家高新区挂牌运作。创成省可持续发展实验区；知识产权示范城市考核在全国地级示范市中排名第四。53个产品获“江苏名牌产品”称号。全市R&D投入占GDP比重为2.55%；高新技术产业产值达4270亿元，增长11.3%；新增国家“千人计划”16人、省“双创计划”68人，新引进创新创业人才团队180个。服务业增加值占GDP比重达46.6%，提高0.5个百分点，旅游业综合收入620亿元、重点景区接待游客1820万人次；物流业增加值、文化产业营业收入分别增长12%和13.5%，惠龙e通建成全国首个标准化大型物流电商平台，睿泰数字出版产业园正式开园。创成镇江国家农业科技园区。粮食生产保持稳定，“农联•亚夫在线”顺利开通，高效设施农业总面积达45万亩，新增5个国家级地理标志农产品和115个无公害、绿色、有机农产品。成功举办海峡两岸名优农产品展销会，交易额和参展人数均创新高。农村水利建设全面加强；实施科学调度、防范在先，有效应对了特大水情。

　　（四）以低碳“镇江模式”走上国际舞台为标志，生态建设跃上新台阶。应邀参加第21届联合国气候变化大会和中美气候领袖峰会，作为全国低碳试点城市的唯一代表，成功举办“城市主题日•镇江”主题边会，为国家低碳建设作出积极贡献，赢得国际社会普遍赞誉；央视《新闻联播》用2分49秒报道了“探索中国低碳发展之路的‘镇江模式’”，新华社、《朝闻天下》、《求是》杂志以及各大媒体都进行了详细报道。“低碳九大行动” 扎实推进，全国首朵“生态云”一期工程上线运行，建成光伏发电项目20个、并网装机容量75.3兆瓦。生态新城建设全面启动。率先实现省级以上园区循环化改造全覆盖，节能考核跻身全省前三。“两个片区”环境综合整治深入推进，西南片区关停污染企业128家，谏壁片区完成重点企业治污项目103个；“一湖九河”水环境综合整治基本完成；太湖水治理考评得分位列全流域第一。实施73项大气污染防治工程，全市空气质量良好以上天数占比达70%，PM2.5平均浓度下降13%。

　　（五）以十大亮点工程全面竣工为标志，城乡面貌发生新变化。连淮扬镇铁路征地拆迁进展顺利，镇江长江大桥开始施工。长江-12.5米深水航道二期工程全面施工。五凤口高架主体完工，312国道镇江城区段改线工程主线贯通，泰州大桥镇江连接线工程全线施工，沪宁高速丹阳东互通建成通车。成为首批国家海绵城市建设试点。解放路南段改造等十大亮点工程全面完成。“7+1”改造加快征收拆迁扫尾，赴上海、厦门城建招商富有成效；市区完成危旧房解危和空斗墙改造10万平方米。建成引航道水利枢纽、焦南闸等水利工程。全市完成293个城市环境综合整治项目，主城区8个街道街巷实现保洁市场化。丹徒区世业镇农村污水管网实现全覆盖。句容市下蜀镇建成美丽宜居镇、戴庄村成为“中国最美休闲乡村”；丹阳市陈山村等10个自然村建成美丽宜居村庄。

　　（六）以在全国率先探索建立“多评合一”制度为标志，深化改革迈出新步伐。生态文明综合改革扎实推进，主体功能区规划深入实施，生态补偿资金、排污权有偿使用等配套政策落实到位。行政审批制度改革取得重要成果，在全国率先实行项目审批“多评合一”和区域评估，审批周期压缩在50个工作日以内，得到国务院调研组的充分肯定，成为全国样板。深化商事制度改革，全面实行 “三证合一”；完成“加强事中事后监管”省级试点工作。机构改革稳步推进，公车改革全面实施。建立市区“多规合一”项目规划审核平台，句容试点工作扎实推进。启动城区土地“六统一”管理和不动产统一登记。四大产业集团公司制改革顺利进行，加快向实体经济转型。组建市级产业发展资金池和新兴产业投资引导基金，“镇科贷”“助农贷”等风险补偿基金运行良好，设立“镇文贷”。5个项目实行PPP运作。全市69.1%的行政村开展农村土地承包经营权确权登记颁证试点工作，扬中市率先全部完成。

　　（七）以十件民生实事如期兑现为标志，群众生活水平获得新提升。特困群体托底保障工程扎实推进，低收入家庭每户每月享受8立方米的免费用气用水，保障力度全省最大。城乡低保标准进一步提高。主城区新建生活无着流浪乞讨人员救助点30个，新建老年人日间照料中心4个、社区助餐点124个。主城区门楼牌整治任务圆满完成。建成征润洲水源地原水水质安全保障工程，金山湖应急备用水源地保护进一步加强，全市人民的饮用水更加安全。完成校安工程69万平方米。创成省级示范乡镇卫生院44个。“绿丝带”行动有效推进，708个村（社区）配备法律顾问。68个基层综合文化服务中心示范点建成运行。“百村万户”双达标行动实现预期目标。新增城镇就业7.93万人，扶持创业5205人，城镇登记失业率稳定在3%以内。扎实做好“东方之星”事件中镇江籍遇难者的善后处置工作，我们对逝者表示沉痛哀悼，愿逝者安息、生者安康！

　　（八）以创成全国文明城市为标志，社会建设开创新局面。以全国第六、全省第一的优异成绩被命名为全国文明城市；“大爱镇江”精神在全市广泛弘扬，赵亚夫被评为全国道德模范，志愿者队伍占总人口比重为全省最高。城乡和谐社区达标率达98%。镇江高校园区展现新姿；市青少年综合实践基地创成国家级，丹阳市、扬中市和润州区跻身全省首批 “义务教育优质均衡发展示范区”；江苏航空职业技术学院获批筹建并顺利通过省级人才培养模式评估。城乡居民大病保险制度全面建立，16家基层医疗卫生机构开设康复联合病房，远程会诊覆盖面进一步扩大。成为全国人口抽样调查唯一综合试点市。认真贯彻中央文艺工作座谈会精神，文艺创作成果丰硕，《茅山小道士》获国家奖项，《花旦当家》获省文华奖一等奖。如期创成“中华诗词之乡”，并实现全国唯一的辖市区“中华诗词之乡”满堂红。成功举办2015“丝路之友”中国—意大利对话会和第七届长江国际音乐节，文化广场标准化建设成为“全国样本”。创成省公共体育服务体系示范区，承办国家乒乓球队争位赛、CBA江苏男篮主场赛、首届镇江国际半程马拉松赛等大型赛事，创成3个国家级航空飞行营地。军民融合发展彰显品牌效应，七创全国双拥模范城顺利通过考核。“六五”普法任务全面完成。全市审计机关开展审计项目225个。海事监管现代化示范区建设取得新成效。安全生产、食品药品安全、社会治安和信访工作全面加强，社会保持和谐稳定。慈善救助实现全覆盖；外事、侨务、民族宗教、档案、地方志、防震、气象、科普等工作取得新成绩，老龄、妇女儿童、关心下一代、残疾人、红十字等事业取得新进展，国家安全、国防教育、人民武装、人民防空等工作取得新成效。援疆援藏工作走在全省前列，南北共建工作积极推进，广大援建干部舍小家为大家，为当地发展作出了积极贡献。

　　（九）以“三严三实”专题教育取得实效为标志，政府自身建设得到新加强。市政府党组扎实开展“三严三实”专题教育，组织6次专题交流研讨，对查摆出的问题逐一整改落实，研究出台财政预算追加、经营性用地容积率调整等多项制度。坚决贯彻市委“三重一大”决策制度，坚持重大事项向市人大常委会报告、向市政协通报，充分发挥各民主党派、工商联、无党派人士以及人民团体的民主监督作用。政府系统承办人大代表建议181件、办理政协提案490件，满意和基本满意率达100%。建立定期学习制度，学法、尊法、用法、守法意识全面增强。成立市政府法律顾问委员会，对政府所有的重大决策、重要协议等进行常态化、合法性审查；出台首部政府规章，推进行政复议规范化。完善政务公开和新闻发布制度，推进行政权力网上公开透明运行。严格执行中央“八项规定”和省委、市委“十项规定”；全面完成年初确定的收费项目“三公开”、治理吃空饷等政府系统16项廉政建设任务，对8起典型案例进行通报曝光，对20个单位、163名责任人进行问责追究。

　　各位代表，去年各项目标任务的完成，标志着“十二五”规划顺利收官！回顾这五年，最大的感受就是：镇江发生了大变化！最大的感慨就是：成绩来之不易！

　　对照“十二五”发展目标，我们交上了一份满意答卷。按照中央要求，全市上下主动认识、适应和引领经济发展新常态，牢牢把握发展主动权，积极作为、攻坚克难。同“十一五”末比，全市地区生产总值增加0.76倍；全社会固定资产投资增加1.5倍；一般公共预算收入增加1.2倍。以高新技术产业为主导、服务经济为主体、先进制造业为支撑、现代农业为基础的现代产业体系格局初步彰显。

　　对照全局和长远发展，我们干成了一批大事要事。率先在全国明确提出碳排放峰值时间，构建低碳管理云平台，项目化开展低碳“九大行动”，低碳城市建设得到习近平总书记的充分肯定。率先在全省建立和实施主体功能区制度，产业集中集聚集约发展由理念变为具体行动，成为全国首批生态文明先行示范区和全省唯一的生态文明建设综合改革试点市，创成国家生态市和国家森林城市。成为苏南国家自主创新示范区的重要一员，创成国家高新区和国家级综合保税区，城市创新力位列全国第19名。奋力作为，成功引进北汽整车制造项目，圆了镇江人28年的汽车梦。

　　对照山水花园城市定位，我们打造了一批特色亮点。五年市区累计完成城建投资2700亿元、拆迁1048万平方米，城市出入口全面整治，西津渡、南山西和北两个出入口成为城市新亮点。建成体育会展中心等一批标志性建筑，打通双井路等一批断头路。各辖市主城建设强势推进、彰显特色。6986个村庄完成环境整治。境内京沪、沪宁、宁杭等高铁相继通车，高铁里程全省最长，阔步迈入高铁时代；建成大路通用机场。荣获中国人居环境奖。

　　对照广大群众期盼，我们办成了一批好事实事。每年集中70%以上的财力用以保障和改善民生。城乡居民人均可支配收入是“十一五”末的1.7和1.8倍；城乡低保标准分别提高60.5%和144%。“一湖九河”“两个片区”以及市区山体整治、南山绿道、“5毛钱公交”、社区助餐点、基层卫生室（院）标准化建设、12870辆公共自行车、100家惠民平价商店等受到群众普遍好评。历经12年努力，成为全国文明城市。公众安全感保持全省前列。

　　各位代表，这些成绩的取得再次有力地证明，有省委、省政府和市委的坚强领导，有市人大、市政协的监督支持，同心同德、励精图治的镇江人完全有信心、有志气不断改变面貌，完全有能力、有办法不断战胜困难，完全有勇气、有干劲不断创造辉煌！

　　在五年的奋斗中，广大基层干部群众吃苦耐劳、埋头苦干；全市各行各业立足岗位、奋力争先；公安干警、驻镇解放军和武警官兵兢兢业业、忠于职守；在镇高校、垂直部门和驻镇单位为镇江发展积极努力、无私奉献；新镇江人热爱镇江、融入镇江；全体人大代表和政协委员胸怀全局、履职尽责；各位老领导老同志满腔热情、心系发展，大家汇聚起了建设新镇江的强大合力！在此，我代表市人民政府，向全市人民、向各界人士、向所有关心支持镇江的海内外朋友们，表示最崇高的敬意和最衷心的感谢！

　　回顾五年来的工作，我们也清醒认识到，发展中还存在一些深层次矛盾和阶段性问题，主要是：生产力布局还需进一步优化；产业转型升级和化解过剩产能的任务仍然繁重，创新驱动的效应还没有充分体现；企业发展面临的困难增加，供给侧结构性改革任重道远，新业态、新技术、新模式还比较少；中心城区的首位度不高，优化配置各类资源的手笔不够大；市场的决定性作用还没有充分发挥，城市开放度还不够高；保障和改善民生仍需做大量工作，基本公共服务均等化体系有待进一步健全；政府治理能力、依法行政水平和干事创业激情有待进一步增强，等等。面对问题和困难，我们决不气馁，一定以对人民、对事业、对城市高度负责的精神，通过艰苦卓绝的工作，让人民更加满意！

二、“十三五”时期的目标任务

　　根据市委六届十一次全会审议通过的《关于制定镇江市国民经济和社会发展第十三个五年规划的建议》，市政府编制了规划纲要（草案），提请大会审议。

　　“十三五”时期，是全面建成小康社会的决胜期，是新常态下转方式、调结构的重要窗口期，也是大有可为的战略机遇期。未来五年，镇江发展的总体要求是：全面贯彻党的十八大和十八届三中、四中、五中全会以及习近平总书记系列重要讲话精神，认真落实“四个全面”战略布局和“五大发展理念”，按照省委十二届十一次全会、市委六届十一次全会的总体部署和要求，以“四基地、一中心、两城市”为总定位，以“稳中奋进”为总基调，以“生态领先、特色发展”为战略路径，同心同德，励精图治，加快建设“强富美高”新镇江。

　　未来五年，我们将认真落实市委全会的决策部署，努力建设“产业特色鲜明、综合实力更强的新镇江，民生加快改善、百姓更加富裕的新镇江，生态品质领先、更加美丽宜居的新镇江，社会充满活力、更加和谐文明的新镇江”。预计全市地区生产总值年均增长8.5%左右；一般公共预算收入力争突破500亿元；固定资产投资年均增长15%；城乡居民人均可支配收入保持与经济增长同步、力争超过；R&D投入占GDP比重达2.8%；在全国率先达到碳峰值，大气、水质等指标走在全省前列，全面完成省下达的节能减排约束性指标。

　　工作中，我们将结合实际，贯彻落实好“五大发展理念”：

　　——崇尚创新，认真解决发展动力问题，推动经济迈向中高端水平。充分发挥科技创新在全面创新中的引领作用，苏南国家自主创新示范区建设取得重大进展，国家级高新区、国家级经济技术开发区在全国排名大幅跃升，成为长江经济带上的重要节点城市。推动大众创业、万众创新，新技术、新产业、新业态蓬勃发展，现代产业体系基本形成。

　　——注重协调，认真解决发展不平衡问题，着力增强发展的整体性。基本公共服务覆盖全部常住人口，户籍人口城市化率达68%左右。加快宁镇扬同城化，努力实现与南京、扬州的轨道交通连接和基础设施互联互通。“两横两纵”的生产力布局基本形成，主城区打造南北贯通的主轴，生态新城展示现实模样；主城与三个辖市的快速通道全部建成，初步形成网络型城市结构；重点中心镇、特色镇加快发展。历史文化的独特魅力充分彰显。

　　——突出绿色，认真解决人与自然和谐问题，建设国家生态文明先行示范区。坚持绿水青山就是金山银山的理念，走生产发展、生活富裕、生态良好的文明发展道路。主体功能区制度全面落实，全市生态和农业空间达70%以上。低碳城市建设的“镇江模式”国际效应更加显著。基本建成城市生态廊道，山更绿、水更清、天更蓝。

　　——强化开放，认真解决发展内外联动问题，全面拓展发展新空间。深度对接“一带一路”、长江经济带战略，推进高水平双向开放，完善法治化、国际化、便利化的营商环境，综合保税区、中瑞生态产业园等建设取得重要进展。建筑、设计、规划、公共资源等市场更加开放有序。行政管理体制、国企国资、农村等各项改革不断深化。

　　——推进共享，认真解决社会公平正义问题，让人民群众有更多的获得感。城乡居民收入持续增加。城市市容更加整洁、出行更加顺畅、管理更加有序。社会保障体系健全，特困家庭通过精准帮扶迈向小康生活。建成苏南教育现代化示范区，现代医疗卫生体系、养老服务体系基本形成，文化体育等设施不断健全，全市生产安全、生活安宁、社会安定。

　　各位代表，实现“十三五”的发展目标，需要全市人民接续奋斗、攻坚克难，团结拼搏、创先争优。我们坚信，当全面建成小康社会之时，当“强富美高”新镇江全面展现之际，作为镇江人，会更加自信、更加骄傲、更加自豪！

三、2016年主要工作

　　今年是实施“十三五”规划的第一年，尽管全球经济增长充满变数，但中国仍处于重要战略机遇期。挑战躲不开、机遇必须抢！只要我们紧紧抓住和充分用好市委全会报告指出的各种机遇，主动认识、适应和引领经济发展新常态，全力稳增长、调结构、惠民生和防风险，就一定能实现“十三五”良好开局！

　　今年经济社会发展的主要预期目标是：继续落实“优势指标保持全省第一方阵、主要指标高于全省平均水平、特色指标力争走在前列”的要求，地区生产总值增长8.5%；一般公共预算收入增长8%左右；固定资产投资增长15%；社会消费品零售总额增长10%以上；R&D投入占GDP比重达2.58%；城乡居民人均可支配收入分别增长8.5%和9%左右；全面完成省下达的节能减排约束性指标。

　　工作中，将紧紧围绕创新转型提速年、产业项目突破年、生态镇江提升年、改革开放深化年、民生保障强化年和治理体系完善年的部署，年初挂图作战、明确责任，年中加强督查、狠抓序时，年底严格考核、兑现承诺。具体抓好六个方面：

　　（一）突出调结构促转型，大力推进产业升级

　　以提高供给体系质量和效率为导向，实施产业升级项目200个，完成年度投资790亿元左右。

　　加快新型工业化步伐。壮大主导产业。高端装备制造业着力做大做强“海陆空”，北汽一期形成实际生产能力；润州船舶与海工配套产业园、镇江新区航空产业园加快建设。新材料产业重点发展高性能合金材料、碳纤维及其复合材料等，推动优势企业进入国家“工业强基工程”专项。培育新兴产业。深入实施《中国制造2025镇江行动纲要》，在生物技术与新医药、新能源、智能电网、新一代信息技术等领域，实现重点突破，引导具备条件的企业进军3D打印及关联材料、石墨烯制备、集成电路等前沿产业，推动惠龙e通等互联网平台企业发展跨界融合，形成新业态，确保新兴产业销售收入增幅高于全省平均水平。提升传统产业。在工业制造和设计领域研究设立市级“工匠奖”；加强品牌和质量建设，支持企业争创国家质量奖。运用新技术、新工艺，改造提升化工、轻工、建材、造纸、纺织等传统产业，全年实施100个重点技术改造项目，技改投入增长19%以上；组织实施20个“两化融合”项目，规模以上企业应用信息技术的比例达92%。淘汰落后和过剩产能。扎实推进“去产能”，综合施策、更多采取兼并重组的方式清理“僵尸企业”，淘汰落后产能生产线40条以上，化解水泥产能100万吨以上，对涉及的相关企业尤其是中小企业，加强政策扶持。

　　提高现代服务业发展水平。认真研究供给侧结构性改革带来的发展机遇，推动形成服务业大发展的热潮。现代旅游业实现综合收入700亿元，重点实施海洋魔幻世界、新河街文化街区、奥悦冰雪馆等项目，办好第六届苏台灯会，支持丹阳“国家旅游产业创新发展实验市”、句容“省乡村旅游发展综合实验区”建设，推进西津渡创建5A级景区，茅山风景区、世业洲积极创建国家级旅游度假区，丹徒长山文化产业园创建省级旅游度假区，打造镇江旅游的特色品牌。整合现有资源，促进旅游与现代农业、文化创意、健康养老、宗教文化等深度融合，构建“大旅游”发展格局。现代物流业完成增加值265亿元，重点整合全市涉港资产，推进江河联动发展，加快实施扬中及镇江新区夹江航道整治、丹阳陵口公用码头建设等工程，规划建设一批挖入式港池，抓实扬中、丹徒、京口等重大物流园区建设，构建长三角区域物流枢纽城市。文化产业实现营业收入600亿元，全面实施“文化+”“互联网+”战略，重点发展创意设计、数字出版、网络游戏、文化旅游四大板块，实施42个重大项目。扶持文化经济实体，加快企业上市步伐。创作一批文艺精品，力争4个以上作品获得省第十二届“五星工程奖”金奖。加快推进体育休闲运动项目，扩大体育消费。

　　推动现代高效农业发展。深化农业结构调整，加快农业“接二连三”步伐，大力发展优质粮油、特色园艺、特种养殖、高效林业、休闲农业五大特色产业。加快国家农业科技园区建设。推动农业适度规模经营，发展生态农业，打造戴庄经验示范村10个，新增市级以上示范家庭农场100家，新型农业经营主体规模经营比重达55%。新增国家级高标准农田3万亩、高效设施农业面积2万亩。大力推进“互联网+现代农业”发展，依托“农联•亚夫在线”，推广“一村一品一店”模式。基本完成土地承包经营权确权登记颁证工作；推进国有林场改革。全面加强农村水利建设，开工建设新孟河延伸拓浚和九曲河整治工程。扎实做好第三次全国农业普查前期准备工作。

　　精准推进产业“三集”。坚持分类指导、提升层次、创造特色，把省级以上开发区、省级现代服务业集聚区作为重中之重，重点推动20个示范园区在完善功能、推进项目、市场运作等方面做出特色、形成效应。按照“一园区、一主导产业、一平台”的思路，打造一批电子商务、金融服务、科技孵化、检验检测等公共服务平台。瞄准世界500强和国内同行业前5强企业，加强项目招引，力争在龙头型项目上形成突破。

　　更高层次扩大双向开放。强化全产业链、精准招商，借助优势企业大力引进境外资金、技术和人才，产业类实际利用外资13亿美元；支持有条件的企业尤其是民营企业“走出去”，提升国际竞争力。国家级综合保税区大力发展保税物流、保税加工、保税服务三大业务，启用“关区内一体化”通关模式，提高通关效率，加强区港联动。中瑞生态产业园加快建设涉外知识产权服务等三大线上平台，新签约项目20个以上。扎实推进海峡两岸新材料产业合作示范区、台江软件园等对台合作八大载体建设，提升项目承载力。争创国家级服务外包示范市。推动发展跨境电子商务，支持丹阳眼镜、家纺与丹徒雪地靴等地方名优产品出口。

　　（二）突出增强发展动力，大力推进创新创业

　　全力推进国家高新区建设。充分发挥在镇高校作用，筹备建设镇江市产业技术研究院，做强江苏科技大学镇江海洋装备研究院。深入推进科技创新一条街建设，打造高创中心、科技大厦等载体；推进镇江知识城建设，构建数字出版技术公共服务等3个平台，吸引30家关联企业入驻。支持扬中加快建设省级高新区，丹阳、句容和丹徒争创省级高新区。

　　加快苏南国家自主创新示范区建设。运用市场化方式，全方位推进“一区十四园”建设。丹阳市高性能合金材料研究院建成测试评价实验中心；句容市高新技术创业园完成二期主体工程，力争创成省级众创空间；扬中市建设智能成套电器试验检测等3个平台，引进5-10个创新创业项目团队；京口区大禹山创意新社区招引企业100家以上；镇江新区新建复合材料研发中心，确保孵化创新企业10家左右。

　　不断提高“双创”能力。围绕全市重点产业，按照“一业一分院”思路，新建省级以上研发机构30家。深入实施“高新技术企业知识产权创造能力提升工程”，推动高新技术企业发明专利全覆盖、大中型企业专利全覆盖。新认定省级高新技术产品200项，高新技术产业产值占规模以上工业产值比重达49%。落实创新创业政策，积极鼓励和引导大众创业，扶持创业7000人，新认定省级以上众创空间10家。

　　强化人才队伍建设。深化人才管理改革试验区建设，采取“项目+人才+资本”的引才模式，实施“金山英才计划”和驻镇高校毕业生留镇三年倍增计划，全年培育引进创新创业领军人才200名、本科以上毕业生1.5万名；加强人才与新兴产业、创新型企业和优势项目的对接，实施万名技能人才培育工程，新增专业技术人才1.8万人，每万人劳动力研发人数达140人。

　　（三）突出打造生态领先城市，大力推进生态文明建设

　　深入推进低碳城市建设。围绕率先成为全国低碳示范城市，拓展联合国气候变化大会、中美气候领袖峰会成果，在低碳技术、低碳能源、低碳交通等领域加强国际合作，申请加入C40；筹办国际低碳技术/产品交易博览会。推进“生态云”二期建设，实现碳排放、污染物排放和资源利用状况看得清、道得明、控得住、管得好、成效显。大力推进“产业碳转型、项目碳评估、企业碳资产、区域碳考核”，当好全国示范。加快推广LED照明、新能源汽车等低碳产品。积极推进扬中绿色能源岛建设，争取在省和国家层面立项，新建分布式光伏发电装机容量40兆瓦；瞄准零碳目标，推进雷公岛开发。构建循环经济体系，积极创建国家可持续发展实验区，扎实推进丹阳国家循环经济示范市建设；加快镇江新区国家级园区循环化改造示范试点，支持丹阳、扬中和句容的开发区争创国家级生态工业园，丹徒开发区争创国家级循环改造示范园区。

　　加快生态新城建设。高标准编制启动区域空间规划并力争获得国家批准，努力成为“国家新型城镇化样板区”。官塘片区完善路网和水系，开工建设凤栖湖；高校园区加快推进，镇江高专、市委党校建成，江大京江学院、江科大新校区主体建筑封顶，完成共享区图书馆和体育馆主体。结合连淮扬镇铁路建设，打造镇江东站综合交通枢纽。推进上湖湿地生态化修复，建设生态绿廊。开展专业化招商，推动一批功能性重大项目签约落户。

　　加强环境综合整治。谏壁片区实施索普集团、谏壁电厂污染治理等20家企业治污工程，扎实推进运河整治、道路建设和居民搬迁；西南韦岗片区加快关停小矿山、小水泥、小石灰窑，大力开展生态修复。“一湖九河”实施水质净化工程并切实加强长效管理，力争主要水体全面达到Ⅲ类水标准。认真做好水土保持、湿地修复等工作，切实保护好长江生态环境。扎实开展新一轮太湖流域治理。加快工矿废弃地恢复治理。强化大气污染防控，狠抓粉尘、尾气、扬尘和油烟等四类专项整治，启动高资片区大气污染整治工程；扎实做好秸秆综合利用和禁烧、工业污染防治等工作，确保全市空气质量良好以上天数全省领先。

　　（四）突出完善功能彰显特色，大力推进城乡建设

　　提升主城功能。加强城市设计。从空间立体性、平面协调性、风貌整体性、文脉延续性等方面，对主城科学规划、严格管控，留住镇江特有“基因”；深入推进市区“多规合一”。改善居住条件。扎实推进房地产“去库存”，全力消化房地产存量，严控新建安置房，把房地产去化周期努力控制在15个月以内。市区完成棚户区、城中村、危旧房等改造91万平方米，有机更新山巷、新河街等片区。完善公共设施。统筹推进103个海绵城市建设项目，建设江苏大学海绵校区，对江滨新村等小区、滨江路等道路实施“低影响开发”改造。南徐片区加快提升商业配套等功能，“三山”景区启动环金山湖景点建设。

　　统筹城乡发展。加快新型城镇化和城乡发展一体化步伐。丹阳重点实施120个城建工程，开工建设357省道，统筹推进城际站、眼镜城片区和练湖片区重点板块建设。句容建成西部干线，实施宁杭高速句容西通道改造，加快建设下蜀、宝华、黄梅、郭庄四个跨界新城。扬中建成新扬南路延伸等道路工程，建设雷公岛直达通道，启动南部新城核心区建设。规划推动丹阳、扬中、镇江新区“金三角”地带跨江联动发展、港产城融合。继续加强重点中心镇建设，推动“两横两纵”沿线和交汇点城镇向小城市发展。完成宝堰、华山等历史文化名镇名村保护发展规划。实施乡村环境卫生提升工程，全面改善农村环境面貌，再建成1个美丽宜居镇、10个美丽宜居村庄。

　　加大城管力度。实施城市管理重心下移，强化属地责任，完善“干—查—督”闭合循环运行机制，研究制定城市综合管理条例。京口、润州所有街道全部实行清扫保洁市场化。实施文明城市创建示范工程，开展城市环境综合整治两年接续行动，立体化整治出新老旧小区、背街小巷和农贸市场。深入开展占道经营、渣土扬尘、户外广告以及违法建设专项整治，继续做好犬类、渣土车管理，对10个老旧小区进行物业提升。再建设一批停车场，缓解市区“停车难”问题。完善数字化城管系统，推动交通信号灯向智能化迈进。切实做好国家卫生城市复审迎检工作。

　　（五）突出优化生产力布局，大力推进基础设施建设

　　构建大交通格局。着力推进宁镇扬同城化。加快连淮扬镇铁路、长江-12.5米深水航道建设，认真研究南京仙林—高资—镇江主城城际轨道、镇江—扬州城市型通道、滨江快速干道等可行方案，争取列入省计划。着力实现城市组团间快速通达。五凤口高架建成通车，丁卯桥路快速化改造启动实施，243省道快速化改造、镇丹高速全面开工；完成苏南运河“四改三”疏浚任务，实现-3.5米水深全线贯通；完成农村公路提档升级180公里。着力完善主城区路网体系。深化有轨电车1号线的研究。加快推进312国道南移、智慧大道南延，改造九华山路，贯通太古山路至长江路，打通矿机路等断头路，提升主城通透性和微循环能力。

　　完善水电气配套。完成大港水厂一期主体工程，加快建设高校园区供水设施，改造集镇内部供水管网18公里；完成征润洲污水处理厂扩建工程，启动丹阳、句容、扬中、丹徒农村污水收集处理设施建设。对城市低压电网和农网进行改造，完成官塘、华山、云台等输变电工程；加快推进丹徒热电联产等项目。改造老小区燃气立管1万户，新建高资燃气高中压站。

　　提升信息基础设施水平。整合各类数据资源，建设镇江市大数据中心。按照“速度最快、收费最低、服务最优”的要求，实施“宽带镇江”工程，光宽带用户占比超过80%，家庭宽带网速20兆以上占比超过60%。实施4G网络无线扩容工程，实现全市4G信号全覆盖。推动主城区以及三个辖市中心城区实现免费wifi。切实加强农村信息基础设施建设。

　　（六）突出满足群众需求，大力推进民生改善

　　保障基本民生。动态调整最低工资标准，引导企业建立职工收入增长与劳动生产率提高相适应的工资增长机制；对茅山老区、相对经济薄弱村和低收入人口实施精准扶贫。认真实施精准帮扶特困家庭奔小康工程。积极扩大就业，新增城镇就业6.5万人，城镇登记失业率控制在3%以内。物价总水平涨幅控制在3%左右。统一城乡居民养老保险和城乡居民医疗保险制度，稳步推进机关事业单位养老保险制度改革，主要险种参保率稳定在98%以上。新增保障性住房3000套，公租房扩大到非户籍人口。全市每千名老人机构养老床位增加到41张。加强未成年人社会保护工作。大力发展慈善和红十字事业。

　　优化公共服务。创建市教育现代化先进镇（街道）10个、先进学校65所；启动特教中心异地新建、江南学校改扩建工程，加快江苏航空职业技术学院建设，力争9月份招生。启动新一轮健康镇江五年行动计划，深化公立医院改革，巩固分级诊疗制度，加快改革药品采购供应体制，加强医疗专业人才队伍建设；整合卫生计生资源，落实全面二孩政策。扎实推进文化惠民，运用PPP模式推进“四馆一中心”建设，建成20个生态文化广场，村（社区）综合文化服务中心实现全覆盖，举办首届镇江金山合唱节，深化“书香镇江”建设；加强文化遗产的保护与利用，市文物安全综合管理信息平台上线运行。启动市体育运动学校建设，承办中国首届科技体育节、国际飞行器设计大赛、中国首届无人机创新大赛等赛事。

　　加强社会管理。进一步完善“网格+网络”社区管理服务体系，全面深化户籍制度改革，实施居住证制度，推动公共服务向居住地转移；完成第十一届村委会和第六届居委会换届选举工作。深化“大爱镇江”建设，壮大志愿者队伍，不断激发社会组织活力。切实加强全民国防教育，巩固双拥“七创”成果，推进军民融合深度发展。创新推进安全生产“八个强化”，防范重特大事故发生。完善食品药品安全监管“161”社会共治模式，争创全国食品安全城市，扬中、丹徒、镇江新区争创首批省级食品安全城市。启动“七五”普法工作，打造市级公共法律服务中心。深化“平安镇江”建设，加强信访基层基础网络建设，全面推进“阳光信访”和“法治信访”；完善立体化社会治安防控体系，建立健全社会矛盾预警机制，依法管理民族宗教事务，营造和谐稳定的社会环境。

四、新常态下善作为

　　适应新常态，需有新状态；引领新常态，更要新作为。面对“十三五”时期的新形势、新任务和新要求，我们将坚持问题导向，进一步转职能、增动能、提效能，做到抓发展上勇担当、新常态下善作为，建设让人民满意的法治型、服务型政府。

　　牢记为民使命。始终把人民群众的需求放在首位，将更多的公共资源向民生倾斜。推进公共服务共建共享，坚持普惠性、保基本、均等化、可持续方向，增加公共服务供给，扩大公共服务范围，创新公共服务方式，积极推进政府购买服务，加快形成政府主导、覆盖城乡、可持续的基本公共服务体系。牢记人民政府为人民，持续改进工作作风，经常走进农村、社区、车间，倾听民声、了解民意，扎扎实实为群众解决问题。充分尊重基层群众的首创精神，团结人民、依靠人民干事创业。

　　善于改革创新。牢固树立不改革就不能前进的强烈意识，继续推进简政放权，深化项目“多评合一”，实施建设领域“多图联审”，研究行政审批集中管理；密切关注“营改增”、消费税等税制改革，全面降低企业和社会交易成本；把地方政府债务纳入统一管理，实施市区土地统一储备、征收、出让。牢固树立不开放就不能生存的强烈意识，用开放的胸襟对待新生事物，更多运用市场机制和办法推动发展。牢固树立不创新就不能升级的强烈意识，深入开展学习型政府建设，不断提高职业水准，在项目建设、招商引资、开展融资等方面，多用新招数，使政府有形之手、市场无形之手、市民勤劳之手同向发力。

　　建设法治政府。全面推进法治政府、法治市场、法治社会一体化建设，使法治成为镇江核心竞争力的重要内容。坚持法定职责必须为，鼓励全体公务人员想干事、敢干事、会干事、干成事，切实做到有作为、敢担当。坚持法无授权不可为，严格按照法定权限和程序行使权力，做到有权不能任性、行权必须依法、用权必受监督、成效社会评价，形成权力闭合运行系统。强化政府信息公开和新闻发布工作，推进公共权力、公共资源、公共服务公开透明。自觉接受人大的法律监督、工作监督和政协的民主监督以及社会公众监督和舆论监督，充分听取民主党派、工商联、无党派人士和各人民团体的意见，确保权力在法治轨道上正确运行。全面加快“诚信镇江”建设，建立失信惩戒机制，形成企业、金融机构、政府和社会人人守信的良好环境。

　　切实廉洁从政。自觉践行“三严三实”，坚持不懈纠正“四风”，努力实现干部清正、政府清廉、政治清明。认真执行中央“八项规定”和省委、市委“十项规定”，巩固“三严三实”专题教育成果，持续加强和改进作风。厉行勤俭节约，反对奢侈浪费，严格控制“三公”经费支出。强化行政监察和审计监督，严格重点领域监管，从源头上预防和治理腐败。认真落实党风廉政建设主体责任和监督责任，严格遵守中国共产党《廉洁自律准则》和《纪律处分条例》，建设风清气正、高效廉洁、人民满意的公务员队伍。

　　各位代表！“十三五”新征程已经开启，全面小康的蓝图已经绘就。让我们始终牢记总书记“镇江很有前途”的殷切希望，在市委的坚强领导下，紧紧依靠全市人民，同心同德、励精图治，向着建设“强富美高”新镇江的宏伟目标奋勇前进!
